# Perceived Thermoregulation Dysfunction and Cardiovascular Symptom Burden in Patients with Metabolic Syndrome: A Multicountry Questionnaire-Based Study

**DOI:** 10.14789/ejmj.JMJ25-0050-OA

**Published:** 2026-01-27

**Authors:** YASHAR MASHAYEKHI, NATHAN KEVIN PETER, SARA BABA-AISSA, SIDRA BAIG, HAMZA BASHEER, NIDA NASIM, ABDULLAH ILYAS, ANN GRACE LAL, MUAZ SHAFIQUE UR REHMAN, FATIMA ASGHAR, SELEEN KAZZAZ, VAREEN KAZZAZ, HALEEMA MAHBOOB AHMAD

**Affiliations:** 1 Department of Orthopaedics, Leicester University Hospitals, Leicester, United Kingdom; 1 Department of Orthopaedics, Leicester University Hospitals, Leicester, United Kingdom; 2 Department of Acute Internal Medicine, Barking, Havering and Redbridge University Hospital NHS Trust, Romford, United Kingdom; 2 Department of Acute Internal Medicine, Barking, Havering and Redbridge University Hospital NHS Trust, Romford, United Kingdom; 3 Department of Internal Medicine, Leicester University Hospitals, Leicester, United Kingdom; 3 Department of Internal Medicine, Leicester University Hospitals, Leicester, United Kingdom; 4 Department of Internal Medicine, Dr VRK Women’s Medical College, Aziznagar, Telangana, India; 4 Department of Internal Medicine, Dr VRK Women’s Medical College, Aziznagar, Telangana, India; 5 Department of Medicine, Kwame Nkrumah University of Science and Technology, Kumasi, Ghana; 5 Department of Medicine, Kwame Nkrumah University of Science and Technology, Kumasi, Ghana; 6 Department of DMOP & General Medicine, Stepping Hill Hospital, Stockport NHS Foundation Trust, Manchester, United Kingdom; 6 Department of DMOP & General Medicine, Stepping Hill Hospital, Stockport NHS Foundation Trust, Manchester, United Kingdom; 7 Department of Acute Medicine, Manchester University Foundation Trust, Manchester, United Kingdom; 7 Department of Acute Medicine, Manchester University Foundation Trust, Manchester, United Kingdom; 8 Department of Medicine, Jinnah Hospital, Lahore, Pakistan; 8 Department of Medicine, Jinnah Hospital, Lahore, Pakistan; 9 Department of Medicine, Ras Al Khaimah Medical and Health Sciences University, Ras Al Khaimah, United Arab Emirates; 9 Department of Medicine, Ras Al Khaimah Medical and Health Sciences University, Ras Al Khaimah, United Arab Emirates; 10 Department of Internal Medicine, University of Sharjah, Sharjah, United Arab Emirates; 10 Department of Internal Medicine, University of Sharjah, Sharjah, United Arab Emirates; 11 Department of General Medicine, University of Sharjah, Sharjah, United Arab Emirates; 11 Department of General Medicine, University of Sharjah, Sharjah, United Arab Emirates; 12 Department of Medicine, Mohammed Bin Rashid University of Medicine and Health Sciences (MBRU), Dubai, United Arab Emirates; 12 Department of Medicine, Mohammed Bin Rashid University of Medicine and Health Sciences (MBRU), Dubai, United Arab Emirates

**Keywords:** metabolic syndrome, thermoregulation, autonomic dysfunction, cardiovascular symptoms, quality of life

## Abstract

**Background:**

Metabolic syndrome (MetS), as a clustering of health disorders, has been linked to the onset of cardiovascular disease. Less is known about how flexible the body is in controlling its own temperature. If this system doesn't work right, it can add stress on the heart and blood vessels.

**Objective:**

To investigate the relationship between symptoms of thermoregulation dysfunction and cardiovascular symptom burden among MetS patients in various countries.

**Methods:**

A cross-sectional questionnaire survey among 603 adult MetS patients from Pakistan, the United Kingdom and the United Arab Emirates. Perceived thermoregulation dysfunction was evaluated with the secretomotor scale of the Composite Autonomic Symptom Score (COMPASS-31), and cardiovascular symptoms were determined with the Seattle Angina Questionnaire (SAQ). Correlations, Mann-Whitney U tests, Kruskal-Wallis tests and multiple regression were used as statistical tests.

**Results:**

A higher score for thermoregulation dysfunctions was associated with lower SAQ scores, reflecting worse HRQL (ρ = -0.350, p < 0.001). Women had more symptoms and worse quality of life compared to men, and older age was associated with dysfunction and worse outcomes (both p < 0.001). The independent predictors of worse SAQ scores in univariate analysis were a higher COMPASS score, older age, female sex, higher body mass index (BMI), comorbidities, and longer disease duration.

**Conclusion**

People with MetS who have difficulty regulating their temperature seem to carry a greater burden of cardiovascular symptoms. Management in these patients may benefit from addressing thermal and autonomic issues.

## Introduction

Metabolic syndrome (MetS) is considered to contribute to the onset of cardiovascular disease, which includes abdominal obesity, insulin resistance, dyslipidemia, hypertension, and systemic inflammation. These can all significantly raise the risk of cardiovascular disease and type 2 diabetes^1, 2)^. Metabolic syndrome prevalence has been on the rise, with 37.6% rising to 41.8% in the past few years^3)^. MetS risk factors were elevated by higher BMI and smoking and by a family history of hypertension or diabetes, and curbed by physical activity and moderate alcohol consumption^4)^.

One underexplored yet physiologically important feature of MetS is the disruption of thermoregulation—the body's inability to maintain a steady-state, optimal temperature in response to environmental or internal thermal insults^5)^. Infrared thermography analysis revealed significant temperature differences between individuals with metabolic syndrome and healthy individuals, with the most important differences observed in the chest and face, reflecting disturbed thermoregulation^6)^.

Changes in thermal sensitivity have been identified as a cause of metabolic syndrome. Increased heat intolerance and reduced sensitivity to cold are associated with a higher prevalence of MetS, regardless of obesity degree. These results indicate that distorted thermal sensation can indicate thermoregulatory and metabolic impairment^7)^.

MetS is also known to be an independent predictor of adverse cardiovascular events, thus significantly increasing the risk of myocardial infarction, stroke, and death in patients with coronary heart disease. Comorbid MetS and high atherosclerotic burden contribute substantially to poor long-term cardiovascular outcomes^8)^. Moreover, long-standing metabolic dysfunction has also been shown to increase CVD risk (including AF), and the cumulative impact of individual MetS components is associated with a substantial increase in overall CVD risk^9)^.

### Rationale

MetS is a significant worldwide health concern, accompanied by rising morbidity and mortality of cardiovascular disease. However, little has been said about the effects it may have on thermoregulation, a process by which the body regulates internal temperature. The abnormalities in vascular tone, metabolic rate, and autonomic control typical of the MetS could impair thermal regulation, leading to heat or cold intolerance and discomfort associated with temperature variations. Such thermoregulatory dysfunctions can even overload the cardiovascular system by altering heart rate, blood pressure, and vascular function. Nevertheless, the perceived state of thermoregulation dysfunction in persons with MetS and its possible connection with cardiovascular symptom burden are little investigated. Thus, a multicountry questionnaire-based study is needed to determine the prevalence of self-perceived thermoregulatory difficulties associated with cardiovascular symptoms among this high-risk population. Knowledge of this relationship may lead to new concepts of patient-centred management methods that consider both metabolic and thermophysiological aspects of health.

### Primary objective:

• To assess the association of cardiovascular symptom burden with perceived thermoregulation dysfunction in metabolic syndrome patients.

### Secondary objectives:

• To determine the prevalence and trend of perceived thermoregulation dysfunction in metabolic syndrome patients in various countries.

• To determine whether the relationship between thermoregulation perception and cardiovascular symptoms depends on demographic and clinical factors (age, sex, BMI, hypertension, and diabetes).

• To compare the burden of cardiovascular symptoms in patients with different levels of perceived thermoregulation dysfunction.

## Materials and Methods

### Methodological design

This was a cross-sectional, multinational, survey-based study evaluating the relationships of perceived thermoregulation dysfunction and CV symptom burden in patients with MetS. A cross-sectional design was chosen to examine, in a short period of time and with limited resources, the associations between self-reported symptoms of impaired thermoregulation and cardiovascular signs.

Respondents were recruited from outpatient clinics, community health centres, and primary care units across multiple countries to represent a wide range of individuals with diverse demographic, socioeconomic, and lifestyle characteristics. Information was gathered on perceived thermoregulation, cardiovascular symptom burden and relevant clinical and lifestyle variables with standardised questionnaires. This approach has allowed an overall assessment of patient-level outcomes, unlike metabolic syndrome, and has withstood scrutiny at multiple sites around the world.

### Sampling technique and sample size

Convenience sampling was used, and all eligible patients with MetS who attended outpatient clinics or health centres in the participating countries during the study period were invited to participate. This method was selected to optimize multisite recruitment and to capture a range of patient experiences regarding thermoregulation and cardiovascular symptoms. Sample size was determined using the World Health Organisation (WHO) formula with a 95% confidence level, a 5% margin of error, and a population proportion of 0.5, assumed to achieve maximum variability in the results. While a minimum sample size of 384 respondents was recommended, 603 respondents were included to achieve better results and account for non-response or incomplete responses.

### Eligibility criteria

They were at least 18 years of age, had metabolic syndrome, and visited one of the participating outpatient clinics, community health centres, or primary care providers during the study period. Respondents attending should be able to read and complete the questionnaire in their native language and provide written informed consent. Exclusion criteria included the presence of recent acute cardiovascular events (myocardial infarction or stroke) within the previous three months, as well as any other severe disease that could influence thermoregulation, such as uncontrolled thyroid disease and end-stage renal failure. Pregnant or breastfeeding women were also excluded due to possible physiological sex differences with respect to thermoregulation.

### Data collection and procedure

A structured, self-administered questionnaire was used to gather data and comprised of three parts: demographic data, clinical characteristics, and standardised assessment tools. Demographic and clinical variables were age, sex, marital status, education, occupation, comorbidities, body mass index, and other factors related to metabolic syndrome. These variables were discussed in relation to the key outcome measures.

Perceived thermoregulation dysfunction was measured with the help of the Secretomotor subscale of the Composite Autonomic Symptom Score (COMPASS-31), which was designed by Sletten et al. in 2012. The COMPASS-31 is a high-quality (31-item) instrument that assesses autonomic symptoms in six domains, specifically the Secretomotor subscale, which gauges sweating abnormalities, heat intolerance, and other thermoregulatory abnormalities. Every item is rated on a Likert-type scale, with higher ratings indicating greater severity of the dysfunction^10)^.

The Seattle Angina Questionnaire (SAQ), developed by Spertus et al. in 1995, was used to measure cardiovascular symptom burden. SAQ is a 19-item tool that measures five domains: physical limitations, angina stability, angina frequency, treatment satisfaction, and quality of life. The domains are rated on a 0-100 scale, with higher scores indicating better health status and fewer symptoms^11)^.

The questionnaires were administered in English, the common medium of instruction and teaching in medicine for the respective countries. Participants received the information in their local language and were assured that they understood it. Researchers also used trained research assistants to help survey respondents in locations such as outpatient and community health centres retrieve their information until they eventually responded. The guidelines regarding written consent, privacy, and ethics were strictly adhered to.

### Statistical analysis

The statistical analyses were performed using IBM SPSS Statistics version 26 (IBM Corp., Armonk, NY, USA). Demographic and clinical characteristics were summarised with descriptive statistics (frequencies and percentages). The boxplots were used to depict the distributions of total Composite Autonomic Symptom (COMPASS) and Seattle Angina Questionnaire (SAQ) scores. Spearman's rank-order correlation was used to evaluate the relationship between COMPASS and SAQ scores. Non-parametric tests were used to analyse group comparisons: the Mann-Whitney U test for gender and the Kruskal-Wallis test for age groups. Factors associated with the total SAQ score, including COMPASS scores and other demographic and clinical measures, were assessed using multiple linear regression. Statistical significance for all analyses was set at p < 0.05.

### Ethical procedures

This study was approved by the Institutional Review Board (IRB) of Manchester University NHS Foundation Trust, Manchester, United Kingdom (IRB Approval No: 2024-REC/0921-MUFT). All participants provided written informed consent after a complete description of the study, including procedures, risks, and benefits. Participation was not mandatory, and withdrawal did not affect medical care. At that point, the data were coded for anonymity and analysed without inclusion of personal identifiers. Data collection was done from September 2024 to December 2024. Participants reporting severe symptoms were advised to seek clinical assessment, consistent with the Declaration of Helsinki.

## Results

### Participant demographics and health profile

The sample (N = 603) was predominantly female (54%) and male (46%), with half of the respondents aged 18-39 years (N = 301), as depicted in Table 1. The majority resided in the UAE (49%), followed by Pakistan (29%) and UK (22%); most were either married (35%) or divorced/separated (32%). The level of education also differed: 31% had a college degree or diploma, and 16% had no education. The participants were primarily students (29%) or homemakers (25%). In terms of health, 32% were obese, 44% were ex-smokers, and 47% said they had moderate physical activity. Type 2 diabetes (34%), dyslipidemia (27%), and hypertension (20%) were the common medical conditions. The most frequent diagnosis of metabolic syndrome was 1-3 years ago (32%), and 42% were on antidiabetic drugs, and 33% were on statins or any other lipid-lowering drugs.

**Table 1:** Demographic characteristics of participants (N = 603)

Variable	f (N)	%		Variable	f (N)	%
Age				Body mass index (BMI)		
18-29 years	170	28		< 25 kg/m^2^ (Normal)	133	21
30-39 years	130	22		25-29.9 kg/m^2^ (Overweight)	161	27
40-49 years	120	20		≥ 30 kg/m^2^ (Obese)	191	32
50-59 years	95	16		Not sure	118	20
60 years and above	88	15		Smoking status		
Gender				Never smoked	248	41
Male	278	46		Former smoker	265	44
Female	325	54		Current smoker	90	15
Country of residence				Physical activity level		
Pakistan	174	29		Sedentary (little or no exercise)	89	15
UK	135	22		Moderate (exercise 1-3 times/week)	286	47
UAE	294	49		Active (exercise ≥4 times/week)	228	38
Marital status				Known medical conditions		
Single	107	18		Hypertension	123	20
Married	209	35		Type 2 diabetes	207	34
Divorced/Separated	195	32		Dyslipidemia (high cholesterol)	165	27
Widowed	92	15		Obesity	67	11
Educational level				None of the above	41	8
No formal education	98	16		Duration of metabolic syndrome diagnosis		
Primary/Secondary school	178	29		< 1 year	133	22
College/Diploma	188	31		1-3 years	195	32
Bachelor's degree	109	18		4-6 years	150	25
Master's degree or higher	30	6		> 6 years	91	15
Occupation				Not officially diagnosed, but have related conditions	34	6
Student	176	29		Current medications		
Employed	37	6		Antidiabetic drugs	255	42
Homemaker	151	25		Statins/Lipid-lowering drugs	198	33
Unemployed	125	21		None	150	25
Retired	114	19				

Note. Values represent frequency (n) and percentage (%) of participants (N = 603)

### Distribution of autonomic symptom burden

**Figure 1:**
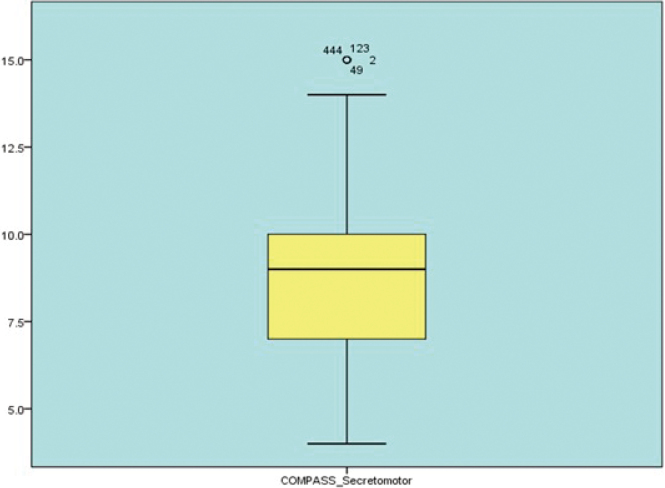
Boxplot of composite autonomic symptom-secretomotor scores (N = 603)

Figure 1 shows the boxplot displaying the distribution of COMPASS (Composite Autonomic Symptom-Secretomotor Score) values. The central line within the box represents the median, while the box edges indicate the interquartile range (IQR), which shows the middle 50% of the data. The whiskers extend to the minimum and maximum values within 1.5 times the IQR. Dots above the whisker indicate outliers, with their case numbers labelled. The yellow box represents the central distribution, and the light blue background is for visual contrast only.

### Distribution of angina-related quality of life

**Figure 2:**
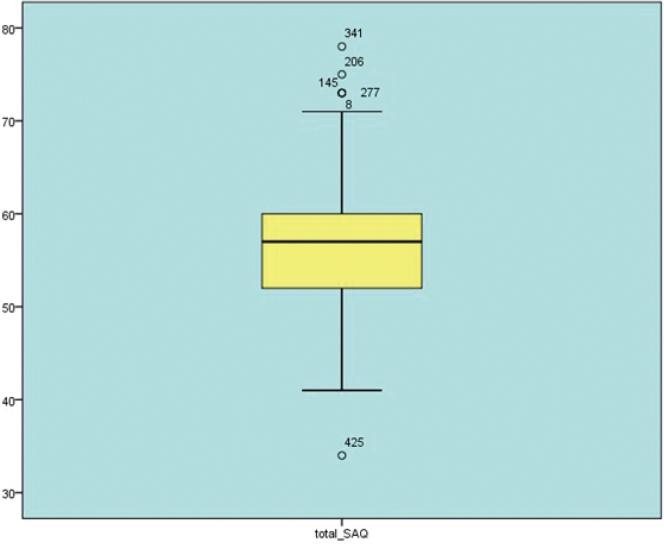
Boxplot of total Seattle Angina Questionnaire (SAQ) scores (N = 603)

Figure 2 shows the box plot illustrating the distribution of total SAQ (Seattle Angina Questionnaire) scores. The central line inside the box represents the median, while the upper and lower boundaries of the box indicate the interquartile range (IQR), which encompasses the middle 50% of the data. The whiskers extend to the minimum and maximum values that are 1.5 times the IQR. Circles outside the range represent outliers and are labelled with their case number. The central portion of the scores is defined in the yellow box, and the blue is only used to differentiate visually.

### Relationship between autonomic symptoms and angina-related QoL

According to Table 2, quality of life related to angina (SAQ) showed a significant negative correlation with the composite Autonomic Symptom-Secretomotor score (r = -0.350, p < 0.001), suggesting that greater autonomic symptom burden was associated with decreased angina-related quality of life in the subjects.

**Table 2:** Spearman's correlations among study variables (N = 603)

Variables	Spearman's ρ	t(df)	p
Composite Autonomic Symptom-Secretomotor	-	-	-
Total Seattle Angina Questionnaire (SAQ)	-0.350	-9.16 (601)	<0.001**

Note. Values are Spearman's rank-order correlation coefficients (ρ); p <0.001 (**), N = 603; All percentages used in descriptive analysis are based on valid responses (i.e., excluding missing data) to ensure consistency across variables.

### Gender differences in autonomic symptoms and angina QoL

There were statistically significant gender differences in the Composite Autonomic Symptom-Secretomotor and angina-related quality of life (SAQ) scores, as shown in Table 3. Female patients reported significantly more autonomic symptoms than male patients (U = 39636, z = -2.78, p = 0.005). In contrast, male patients had higher angina-related quality- of-life (SAQ) scores than female participants (U = 39052, z = -2.42, p = 0.016).

**Table 3:** Mann-Whitney U tests comparing gender differences in subscale of Composite Autonomic Symptom-Secretomotor and total Seattle Angina Questionnaire (SAQ) scores among participants (N = 603)

Variable	Gender	N	Mean rank	Sum of ranks	U	Z	p
Composite Autonomic Symptom-Secretomotor	Male	278	276.45	76,836.00	39,636.000	-2.78	0.005**
	Female	325	323.51	105,641.00			
Total Seattle Angina Questionnaire (SAQ)	Male	278	328.1	91,212.00	39,052.000	-2.42	0.016*
	Female	325	279.74	90,638.00			

Note. N = 603 (Males = 278, 46%; Females = 325, 54%); Mann-Whitney U test was used for all comparisons; p values marked with *, ** indicate statistical significance at p < 0.05, < 0.01

### Age-related variation in autonomic symptoms and angina QoL

Table 4 shows that both Composite Autonomic Symptom-Secretomotor and angina-related quality of life (SAQ) differed significantly by age category. Older participants also had higher autonomic symptom scores, with those over 60 years reporting the highest mean rank (372.6), while the lowest was among younger adults (18-29: 245.3) (χ^2^ (4) = 24.39, p < 0.001). In contrast, the reduction in angina-related QoL was age-dependent, and the oldest group had significantly lower SAQ scores (mean rank = 238.4) than the youngest group (mean rank = 348.9) (χ^2^ (4) = 20.15, p < 0.001).

**Table 4:** Kruskal-Wallis tests comparing subscale of Composite Autonomic Symptom-Secretomotor, and Seattle Angina Questionnaire (SAQ) Scores across age groups (N = 603)

Variable	Age group	N	Mean rank	*x*^2^(*df* = 4)	p
Composite Autonomic Symptom-Secretomotor	18-29 years	170	245.3	-	-
	30-39 years	130	278.45	-	-
	40-49 years	120	312.1	-	-
	50-59 years	95	347.85	-	-
	60 years and above	88	372.6	24.39	< 0.001***
Total Seattle Angina Questionnaire (SAQ)	18-29 years	170	348.9	-	-
	30-39 years	130	315.25	-	-
	40-49 years	120	286.7	-	-
	50-59 years	95	262.15	-	-
	60 years and above	88	238.4	20.15	< 0.001***

Note. N = number of participants in each age category; % = percentage of the total sample; Percentages are based on total N = 603; Values are mean ranks from Kruskal-Wallis H tests; Overall test statistics are reported in the bottom row for each variable: COMPASS-Secretomotor (χ^2^(4) = 24.39, < 0.001***), and Total SAQ (χ^2^(4) = 20.15, < 0.001***); Significance levels: p < 0.001***

### Predictors of angina-related quality of life

As presented in Table 5, multilinear regression analysis indicated that higher Autonomic Symptom-Secretomotor scores, older age, female gender, greater BMI, comorbid conditions, longer duration of metabolic syndrome, and medication use were significant predictors of lower angina-related quality of life (SAQ) scores (all p ≤ 0.006). Of these, age (β = -0.204, p < 0.001) and known disease history (β = -0.201, p < 0.001) were most strongly negatively correlated with COMPASS scores, and each point increase in COMPASS score was associated with a 0.485-point decrease in SAQ scores (p < 0.001).

**Table 5:** Multiple linear regression predicting total Seattle Angina Questionnaire (SAQ) from clinical and demographic factors (N = 603)

Predictor	B	SE	β	t	Sig.	95% Cl LL	95% Cl UL
Constant (total Seattle Angina Questionnaire)	72.184	2.345	-	30.78	< 0.001***	67.580	76.790
Composite Autonomic Symptom-Secretomotor	-0.485	0.096	-0.182	-5.05	< 0.001***	-0.674	-0.296
Age	-0.932	0.212	-0.204	-4.40	< 0.001***	-1.350	-0.514
Gender (1 = male, 2 = female)	-3.865	1.128	-0.148	-3.43	0.001**	-6.083	-1.647
BMI	-0.527	0.187	-0.119	-2.82	0.005**	-0.895	-0.159
Known medical conditions	-1.074	0.265	-0.201	-4.05	< 0.001***	-1.596	-0.552
Duration of metabolic syndrome diagnosis	-0.663	0.241	-0.123	-2.75	0.006**	-1.136	-0.190
Current medications	-0.586	0.203	-0.112	-2.89	0.004**	-0.985	-0.187

Note. N = 603, B = unstandardized regression coefficient; SE = standard error; β = standardised regression coefficient; CI = confidence interval; LL = lower limit; UL = upper limit. All predictors were entered; p < 0.01**, p < 0.001***.

## Discussion

This paper examined the relationship between perceived thermoregulation dysfunction and cardiovascular symptom burden in people with metabolic syndrome (MetS) in three countries. In our research, the higher the secretomotor symptom scores, the lower the SAQ scores, indicating poorer quality of life related to angina. This is substantiated by evidence of exaggerated blood pressure responses to thermal variations in persons with cardiovascular comorbidities, and by the importance of autonomic/secretomotor dysfunction in cardiovascular strain^12)^.

Females had greater secretomotor symptom scores and reduced SAQ scores than males, suggesting worse angina-related quality of life. These results are in line with previous studies suggesting that women suffer more severe autonomic dysfunction and worse quality of life than men^13, 14)^.

Older patients (adults ≥ 60 years) had significantly higher secretomotor scores and lower SAQ scores, indicating more severe autonomic problems, as well as worse angina-related quality of life with advancing age. This is consistent with prior studies that have shown elderly patients, even in the absence of myocardial infarction (MI), to have increased autonomic symptoms and lower baseline SAQ scores^15, 16)^.

In multiple linear regression, greater reporting of secretomotor symptoms, female gender, and older age were independently associated with lower SAQ scores, indicating that these factors are strong contributors to worse angina quality of life^12, 14, 16)^. Additionally, higher BMI and comorbidity were independently associated with lower SAQ scores, suggesting the deleterious effect of obesity as a clinical/lifestyle condition on quality of life. These results are consistent with previous studies showing lower physical function and quality of life in overweight/obese CAD patients^17)^, lower SAQ scores in older adults with chronic conditions or history of CABG^18)^; worse HRQoL among individuals with longer-standing cardiovascular disease or metabolic conditions^19)^ as well as worse quality of life and higher frailty among older people using polypharmacy^20)^.

Collectively, our findings highlight the multifactorial nature of angina and quality of life in patients with metabolic syndrome, and the need to address better autonomic dysfunction, gender-specific differences in the heart rate response profile, age-related decline in fitness performance and comorbid cardiovascular disease, such as obesity and medication burden.

This study has several limitations. First, the cross-sectional nature of this study limits causal inference and finalises the direction of causality between thermoregulation dysfunction and cardiovascular symptom volume. Second, the convenience sampling we used also limits the generalizability of our study findings, as these samples may not fully represent the larger MetS population with diverse socioeconomic and climatic backgrounds. Third, the self-report nature of COMPASS-31 and SAQ makes them vulnerable to recall and response bias, potentially affecting the accuracy with which participants characterise their symptoms. Furthermore, no objective physiological measurements of thermoregulation (e.g., skin temperature mapping or sweat rate) or autonomic function (e.g., heart rate variability) were used, which could have supported the interpretation of the results. Whether thermal perception was influenced by environmental conditions, i.e., ambient temperature or humidity, could not be ruled out.

## Conclusion

In conclusion, in this multicountry study, perceived thermoregulation dysfunction was significantly associated with cardiovascular symptom burden among METS patients. Greater secretomotor autonomic dysfunction was associated with worse QoL as it pertains to angina, and particularly so among older individuals and women. These results highlight the interrelationship among autonomic dysfunction, thermal regulation and cardiovascular health in metabolic diseases. Considering thermophysiological parameters, rather than only traditional metabolic factors, might further optimise personalised management of MetS and cardiovascular prevention in individuals with MetS.

## Author contributions

YM conceived and designed the study and supervised the overall project. NKP contributed to the study design, data interpretation, and manuscript editing. SBA assisted in data acquisition and coordination across study sites. SB contributed to literature review, data collection, and manuscript drafting. HB participated in clinical data analysis and verification of results. NN and AI were responsible for statistical analysis, data entry, and quality assurance. AGL coordinated the study, oversaw data collection in all regions, and served as the corresponding author. MSR facilitated patient recruitment and collection of clinical information in Pakistan. FA assisted with data organization and literature synthesis. SK and VK contributed to the review of results and proofreading of the final manuscript. HMA assisted in data compilation and formatting of tables and figures. All authors read and approved the final manuscript.

## Conflicts of interest statement

The authors declare that there are no conflicts of interest.
